# Characteristics of in peripheral blood of 70 hospitalized patients and 8 diarrhea patients with COVID-19

**DOI:** 10.7150/ijms.45743

**Published:** 2020-05-17

**Authors:** Xiao-Yu Wei, Ding Jing, Bei Jia, Qing Li, Xia-Qia Zhou, Ming-Feng Gong, Jing-Bo Zou, Qiang Zhang, Wen-Xiang Huang, Wen-Guang Tian

**Affiliations:** 1 Department of Infectious Diseases, Yongchuan Hospital of Chongqing Medical University, Chongqing 400016, P.R. China.; 2 Cancer center, Daping Hospital, The Third Military Medical University, Yuzhong District, Chongqing 400042, P.R. China.; 3 Department of Infectious Diseases, The First Affiliated Hospital of Chongqing Medical University, Chongqing 400016, P.R. China.; 4 Chongqing Yongchuan District Center for Disease Control and Prevention, Chongqing 400016, P.R. China.

**Keywords:** Peripheral blood, COVID-19, GGT, Diarrhea

## Abstract

**Objective:** To analyze the blood test indicators of patients after infection of COVID-19 in Chongqing and analyze the clinical indicators of 8 patients with diarrhea.

**Materials and Methods**: From January 26, 2019 to February 13, 2020, 70 patients diagnosed with 2019-nCoV according to the World Health Organization interim guidance for NCP and divided into diarrhea and non-diarrhea groups. The laboratory tests liver and kidney function, blood routine, coagulation function, and immune status.

**Results**: The study population included 70 hospitalized patients with confirmed CONV-2019. NCP patients (43males and 27 females) with a mean age of 48.57±17.80 (9~82) years and only 4.3% of patients have lung-related diseases. The positive rate of ESR, CRP, PT, IL6, lymphocyte count, GGT, Prealbumin and CD4 was more than 50%. We further analyzed the differences between 8 diarrhea patients and 62 non-diarrhea patients. Among these indicators, only Lymphocyte, CRP, Prealbumin and Cystatin C positive rate is more than 50%. Although there is no statistical difference in GGT, 100% of the 7 patients tested decreased.

**Conclusion:** Our data recommended that the ESR, CRP, PT, IL6, lymphocyte count, GGT, prealbumin and CD4 have important value in the diagnosis of COVID-19, and the decrease of GGT may be an important indicator for judging the intestinal dysfunction of patients.

## Introduction

The new coronavirus (CoV) named by the World Health Organization (WHO) as “COVID-19” led to a pneumonia outbreak that recently started in Wuhan, Hubei Province China 1. Its outbreak was linked to a large seafood and animal market and investigations are underway to determine the source of infection. Thousands of human infections and many export cases worldwide have been confirmed in China to date 2. The main source of infection is pneumonia in patients infected with new coronavirus. Respiratory droplets are the main route of transmission and can also be transmitted through contact. People are often vulnerable. The main symptoms are respiratory symptoms such as fever and cough. However, recently, Professor An Ping et al. observed that the first symptom of some patients with pneumonia infected by the new coronavirus is only diarrhea, and it is suspected that the digestive system may also transmit the new coronavirus. At present, the mechanism by which digestive symptoms appear is unclear. The purpose of this study was to analyze the basic conditions of 70 patients with new-type coronavirus in Yongchuan Hospital, to analyze the blood test indicators of patients after infection, and the differences in the detection indexes of the infected people in Wuhan and Chongqing, and to analyze the clinical indicators of 8 patients with diarrhea. This will assist in the ability to find specific clinical testing indicators for patients with diarrhea.

## Materials and Methods

### Patients

From January 26, 2019 to February 13, 2020, 70 patients diagnosed with 2019- nCoV according to the World Health Organization interim guidance for NCP and divided into diarrhea and non-diarrhea groups. All patients were confirmed to be positive for new coronavirus nucleic acid by real-time fluorescent RT-PCR. Infection samples were collected from Chongqing Hospital.

### Blood sampling

Blood samples of the patients were collected by the nurse according to the doctor's order, and all patients were not treated before the blood sampling or did not receive the standardized treatment according to the diagnosis and treatment scheme of NCP.

### Blood test

Liver and kidney function, immunoglobulin complement, and fructosamine machines are all Hitachi 7600 automatic biochemical analyzers from Japan. The blood routine is a Japanese Sysmex XN-1000 automatic blood analyzer. The coagulation test is a Japanese Sysmex CA7000 automatic coagulation analyzer. Glycated hemoglobin is a Mindray H50 glycated hemoglobin analyzer. Erythrocyte sedimentation is an instrument that I haven't noticed, and I haven't changed it yet.

### Statistical analysis

All statistical analyses were performed using SPSS 20.0 (SPSS Inc., Chicago, IL, USA). Descriptive analyses were performed for categorical variables such as gender. Continuous variables such as inspection results were expressed as x ± s and compared using the independent samples t-test. P<0.05 was considered as statistically significant.

## Results

### Clinical characteristics of all patients

All patents' clinical specimens for CONV-2019 diagnostic testing were obtained in accordance with the guidelines of the CDC 3,4. A description of the assay and sequence information for the RT-PCR primers and probes are available on the CDC Laboratory Information website for CONV-2019.

The study population included 70 hospitalized patients with confirmed CONV-2019. NCP patients (43males and 27 females) with a mean age of 48.57±17.80 (9~82) years and only 4.3% of patients have lung-related diseases. Lesion site locations were prominent in both lungs (Table 1).

### Blood test indicators of all patients

All patients were tested for blood routine, liver and kidney function, coagulation, and immune status when they were admitted to the hospital. ESR(39/70), CRP (49/70), PCT (31/58), PT (41/67), IL6 (39/68), Cystatin C (23/68) were significantly elevated and the lymphocyte count (39/70), Prealbumin (39/67), GGT (54/70), CD4 (50/66), CD8 (24/70), T cell (26/65) were significantly decreased and the abnormal rate was more than 30%. At the same time, the positive rate of ESR, CRP, PT, IL6, lymphocyte count, GGT, Prealbumin and CD4 is more than 50%. These indicators may have more important clinical significance in the diagnosis process (Table 2).

### Comparison of diarrhea and non-diarrhea populations

**Figure 1:**
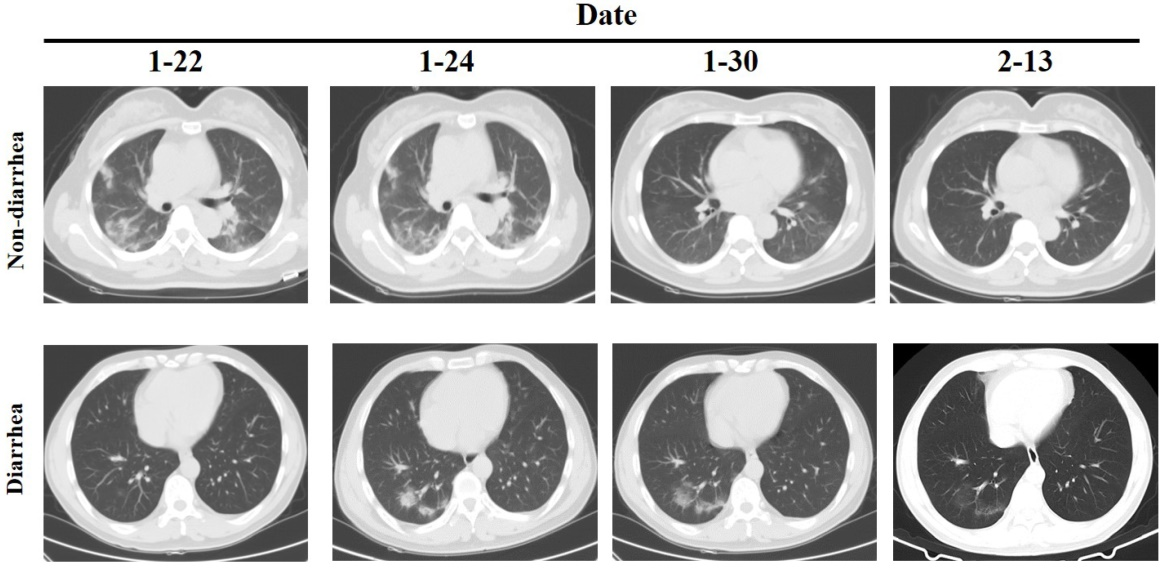
CT of chest in patients with diarrhea and non-diarrhea.

However, the clinical examination of patients with diarrhea as the main symptom is not clear. We further analyzed the differences between 8 diarrhea patients and 62 non-diarrhea patients. The Eight patients had diarrhea as the first symptom, and the throat swab test was negative but the anal swab test was positive, and except for one patient who had slight inflammation of the lungs when admitted, the other patients had no obvious inflammatory changes in the lungs (Figure 1). There was no significant difference between the two groups in the general situation of age, gender, medical history, etc. (Table 3). Comparison of clinical test indicators between the two groups of patients found that GGT (diarrhea *vs.* non-diarrhea: 20.75±14.12 *vs*. 54.74±59.61; P=0.000) was significantly different. The results indicate that GGT may be a deliberate detection index for patients with diarrhea. Further analysis of clinical test indicators of 8 patients showed that the Lymphocyte (4/8), PLT(2/8), CRP (4/8), PT (2/8), Prealbumin (5/8), Creatinine (2/8), Cystatin C (4/8), C4 (3/8) and CD8 (3/8) had significant differences (P<0.05). Among these indicators, only Lymphocyte, CRP, Prealbumin and Cystatin C positive rate is more than 50%. Although there is no statistical difference in GGT, 100% of the 7 patients tested decreased, so it further illustrates the important detection value of GGT in patients with diarrhea.

## Discussion

So far, there is no vaccine or specific antiviral treatment for this emerging infectious disease, and effective control still depends mainly on early diagnosis, patient isolation and close contact monitoring 3. Therefore, reliable and accurate diagnostic methods play a vital role in the field of disease control and prevention. However, some patients with diarrhea have a false-negative nucleic acid test. So, finding indicators that can predict gut symptoms in patients has important clinical significance. Our data found that the positive rate of ESR, CRP, PT, IL6, lymphocyte count, GGT, Prealbumin and CD4 was more than 50%. In addition, it was found that the Lymphocyte, PLT, CRP, PT, Prealbumin, Creatinine, Cystatin C, C4 and CD8 have clinical significance for patients in the diagnosis of diarrhea. GGT reduction is probably the most important indicator. Taken together, consistent with previous research 2,5, the ESR, CRP, PT, IL6, lymphocyte count, GGT, Prealbumin and CD4 have important value in the diagnosis of COVID-19, and the decrease of GGT may be an important indicator for judging the intestinal dysfunction of patients.

## Clinical Perspectives

Our study analyzed the detection of 70 patients with COVID-19 infection in Chongqing, and specifically analyzed the blood test of 8 patients with diarrhea as the first symptom. The decrease of GGT may be an important indicator for judging the intestinal dysfunction of patients. It has clinical significance for the diagnosis and treatment of diarrhea patients.

## Figures and Tables

**Table 1:** Case characteristics group by 2019 ncov

Variable	Patient Number	Patient%
Age (mean±SD)	48.57±17.80	
**Age(y)**		
<60	52	74.3
≥60	18	25.7
**Gender**		
Male	43	61.4
Female	27	38.6
**Lung related basic diseases**		
no	67	95.7
yes	3	4.3
**Person category**		
A	29	41.4
B	41	58.6
**Location of lesion**		
Right	10	14.3
Left	7	10.0
Both	46	65.7
Normal	7	10.0

A: Wuhan infected people; B: Infecting people in Chongqing.

**Table 2:** Differences in clinical blood test indicators among all patients

Variable	Normal NO. (mean±SD)	High NO. (mean±SD)	Low NO. (mean±SD)	P value (mean)
WBC	59 (5.35±1.53)		11 (2.90±0.53)	b, *P*=0.000
Lymphocyte	31 (1.52±0.36)		39 (0.76±0.23)	b, *P*=0.000
Neutrophil cell	63 (3.5±1.09)	3 (7.55±3.61)	4 (1.23±0.28)	a, *P*=0.000 b, *P*=0.000
N% (40-75)	57 (64.81±8.56)	13 (81.68±6.20)		a, *P*=0.000
PLT	55 (195.56±51.03)	3 (325±8.49)	12 (95.75±19.07)	a, *P*=0.000 b, *P*=0.000
HB	54 (105.37±9.10)	8 (157.27±6.25)	8 (133.39±9.39)	a, *P*=0.000 b, *P*=0.000
CRP	31 (3.11±2.82)	39 (36.26±27.79)		a, *P*=0.000
ESR	21 (10.26±4.25)	49 (60.80±29.36)		a, *P*=0.000
PCT	27 (0.06±0.03)	31 (0.12±0.17)		a, *P*=0.041
IL6	29 (2.66±1.58)	39 (23.91±19.80)		a, *P*=0.000
BNT	63 (76.13±83.57)	5 (500±320.20)		a, *P*=0.041
PT	26 (12.07±0.66)	41 (14.69±1.39)		a, *P*=0.000
PTA	62 (88.10±8.69)	6 (61.83±2.40)		a, *P*=0.000
d-dimer	59 (0.35±0.23)	3 (1.30±0.17)		a, *P*=0.000
ALT	62 (24.03±9.23)	8 (89.75±37.72)		a, *P*=0.002
AST	55 (25.07±5.90)	15 (56.73±29.20)		a, *P*=0.001
GGT	9 (83.67±27.69)	7 (194.86±51.04)	54 (26.72±18.57)	a, *P*=0.001 b, *P*=0.000
Prealbumin	28 (242±28.17)		39 (131.51±41.87)	b, *P*=0.000
ALB	58 (42.33±4.11)		11 (33.38±1.42)	b, *P*=0.000
CK	51 (66.67±30.20)	11 (14.64±49.22)		b, *P*=0.008
CK-MB	53 (0.46±0.45)	9 (5.97±2.05)		a, *P*=0.043
LDH	57 (470.37±96.41)	7 (749.59±124.89)		a, *P*=0.001
Myoglobin	60 (31.05±14.61)	8 (313.45±584.95)		a, *P*=0.214
Creatinine	60 (67.62±13.23)	2 (128±8.49)	8 (38.75±3.85)	a, *P*=0.000 b, *P*=0.000
Urea nitrogen	60 (4.53±1.17)		9 (2.52±0.29)	b, *P*=0.000
α1 microglobulin	57 (21.84±4.57)	11 (34.69±4.44)		a, *P*=0.000
Cystatin C	45 (0.95±0.11)	23 (1.34±0.32)		a, *P*=0.000
Glycated hemoglobin	40 (5.74±0.63)	10 (8.14±2.80)		a, *P*=0.027
IgG	52 (11.62±2.06)	10 (18.49±3.12)	2 (7.2±0.85)	a, *P*=0.000 b, *P*=0.004
IgA	55 (1.95±0.53)	9 (4.53±1.27)		a, *P*=0.000
IgM	61 (1.22±0.43)	2 (2.44±0.08)	1 (-)	a, *P*=0.000
C3	62 (1.32±0.17)	2 (1.75±0.05)		a, *P*=0.001
C4	47 (0.31±0.06)	15 (0.48±0.10)	2 (0.16±0.01)	a, *P*=0.000 b, *P*=0.001
CD4	16 (715.69±161.46)		50 (314.78±126.11)	b, *P*=0.000
CD8	46 (365.47±175.03)		24 (126.21±39.04)	b, *P*=0.000
T cell	39 (917.44±333.36)		26 (403.31±180.52)	b, *P*=0.000

Note: a: high vs normal, b: low vs normal.

**Table 3:** Comparison of clinical data between diarrhea and non-diarrhea

Variable	Diarrhea (n=8)	Non-diarrhea (n=62)	P value
Age (mean±SD)	42.38±17.74	49.37±17.80	0.299
**Age(y)**			
<60	8	43	0.097
≥60	0	19	
**Gender**			
Male	5	39	0.714
Female	3	23	
**Lung related basic diseases**			
no	8	59	0.771
yes	0	3	
**Location of lesion**			
Right	0	12	0.420
Left	0	4	
Both	1	38	
Normal	7	8	

**Table 4:** Comparison of clinical indicators of diarrhea and non-diarrhea

Variable	Diarrhea	No diarrhea	P value
WBC	4.50±1.18	5.21±2.08	0.353
Lymphocyte	1.17±0.49	1.08±0.50	0.635
Neutrophil cell	2.96±1.35	3.61±1.80	0.327
N%	63.25±15.19	67.84±10.30	0.266
PLT	182.63±46.88	180.81±66.96	0.941
HB	132.50±13.94	134.13±16.46	0.790
CRP	22.36±23.17	21.48±27.10	0.931
ESR	41.50±47.48	47.60±32.05	0.635
PCT	0.22±0.36	0.07±0.04	0.276
IL6	11.55±21.95	15.06±17.94	0.614
BNT	73.63±83.54	98.9±141.59	0.624
PT	12.93±1.60	13.74±1.84	0.241
PTA	87.65±7.21	83.67±16.34	0.502
d-dimer	0.32±0.24	0.41±0.31	0.456
ALT	21.13±17.46	32.89±26.43	0.227
AST	27.00±16.54	32.48±19.63	0.453
**GGT**	**20.75±14.12**	**54.74±59.61**	**0.000**
Prealbumin	169.63±73.49	178.7±65.47	0.716
ALB	42.21±5.84	40.73±4.95	0.438
CK	67.63±40.13	117.04±150.14	0.361
CK-MB	0.34±0.20	0.81±1.44	0.364
LDH	521.38±189.60	532.76±169.68	0.861
Myoglobin	27.49±8.54	69.18±223.90	0.603
Creatinine	62.86±17.04	66.45±19.06	0.615
Urea nitrogen	3.98±1.05	4.39±1.46	0.446
α1 microglobulin	23.13±6.27	24.03±6.65	0.718
Cystatin C	1.02±0.15	1.09±0.28	0.510
Glycated hemoglobin	5.81±0.30	6.15±1.98	0.676
IgG	11.77±1.95	12.65±3.61	0.530
IgA	2.62±1.36	2.30±1.09	0.484
IgM	1.57±0.55	1.21±0.46	0.061
C3	1.29±0.12	1.34±0.19	0.506
C4	0.37±0.05	0.34±0.11	0.557
CD4	404.63±153.05	431.19±265.49	0.784
CD8	319.50±210.50	274.37±179.99	0.516
T cell	764.13±349.30	733.25±438.44	0.849

**Table 5:** Analysis of clinical detection indicators in patients with diarrhea

Variable	Normal NO. (mean±SD)	High NO. (mean±SD)	Low NO. (mean±SD)	P value
WBC	7 (4.75±1.00)			
**Lymphocyte**	**4 (1.51±0.29)**		**4 (0.83±0.39)**	**a, *P*=0.032**
Neutrophil cell	6 (3.43±1.21)		2 (1.53±0.21)	a, *P*=0.081
NEU	7 (59.03±10.15)	1(92.8)		
**PLT**	**6 (204.67±27.19)**		**2 (116.50±4.95)**	**b, *P*=0.005**
HB	4 (135.57±11.77)			
**CRP**	**4 (2.60±2.73)**	**4 (42.13±14.29)**		**a, *P*=0.010**
ESR	5 (12.2±6.22)	3 (90.33±45.72)		a, *P*=0.096
PCT	4 (<0.05)	4 (0.40±0.47)		a, *P*=0.235
IL6	6 (<1.5)	3 (34.44±25.14)		a, *P*=0.151
BNT	8 (73.63±83.54)			
**PT**	**6 (12.15±0.83)**	**2 (15.25±0.35)**		**a, *P*=0.003**
PTA	8 (87.65±7.24)			
D-dimer	8 (0.32±0.24)			
ALT	7 (15±2.31)			
AST	7 (21.43±5.44)			
**GGT**			**7(15.86±3.02)**	
**Prealbumin**	**3 (248.33±28.10)**		**5 (122.40±40.30)**	**a, *P*=0.003**
ALB	7 (43.26±5.44)			
CK	7 (54.57±16.99)			
CK-MB	8 (0.34±0.20)			
LDH	6 (423.83±61.75)	2 (814±65.05)		
Myoglobin	8 (27.49±8.54)			
**Creatinine**	**6 (71.00±9.23)**		**2 (38.5±4.95)**	**b, *P*=0.004**
Urea nitrogen	7 (4.27±0.71)			
α1 microglobulin	7 (21.53±4.69)			
**Cystatin C**	**4 (0.9±0,09)**	**4 (1.15±0.06)**		**a, *P*=0.005**
Glycated hemoglobin	6 (5.81±0.30)			
IgG	6 (12.2±1.74)			
IgA	5 (1.93±0.48)	2 (4.34±1.14)		a, *P*=0.237
IgM	6 (1.41±0.41)	1 (2.5)		
C3	7 (1.23±0.12)			
**C4**	**5 (0.34±0.03)**	**3 (0.43±0.01)**		**a, *P*=0.017**
**CD4**	**4 (509.05±9.00)**		**4 (340.80±165.40)**	**b, *P*=0.084**
**CD8**	**5 (429.20±189.63)**		**3 (136.67±54.27)**	**b, *P*=0.044**
T cell	7 (855.14±255.00)		1 (127)	

Note: a: high vs normal, b: low vs normal.

**Table 6:** Test indicator description and normal value range

Blood routine	
Leukocytes (×10^9^ per L, normal range 3.5-9.5)	Lactate dehydrogenase (U/L, normal range 313-618)
Neutrophils (×10^9^ per L, normal range 1.8-6.3)	Myoglobin (ng/mL, normal range Male 0-121; Female 0-61.5)
Neutrophil percentage (Neutrophil percentage 40-75)	Serum Creatinine (umol/L, normal range Male 53-123; Female 62-106)
Lymphocytes (×10^9^ per L, normal range 1.1-3.2)	Blood Urea nitrogen (mmol/L, normal range 2.86-8.20)
Platelets (×10^9^ per L, normal range 125-350)	α1 microglobulin (mg/L, normal range 10-30)
Hemoglobin (g/L, normal range Male 130-175; Female 115-150)	Cystatin C (mg/L, normal range 0.51-1.09)
Brain natriuretic peptide (pg/ml, normal range 1-125)	Glycated hemoglobin (%, normal range 4.5-6.3)
Coagulation function	Immunoglobulin complement G (G/L, normal range 8-16)
Prothrombin time (s, normal range 11-14.5)	Immunoglobulin complement A (G/L, normal range 0.7-3.3)
D-dimer (ug/L, normal range 0.00-0.5)	Immunoglobulin complement M (G/L, normal range 0.5-2.2)
Prothrombin time activity (%, normal range 70-120)	Complement 3 (G/L, normal range 0.8-1.6)
Blood biochemistry	Complement 4 (G/L, normal range 0.2-0.4)
Alanine aminotransferase (U/L, normal range Male 9-50; Female 7-40)	Infection-related factors
Aspartate aminotransferase (U/L, normal range Male 15-40; Female 13-35)	Erythrocyte sedimentation rate (mm/h, normal range Male 0-15; Female 0-20)
Gamma glutaminase (GGT) (U/L, normal range Male 10-60; Female 7-45)	C-reactive protein (mg/L, normal range 0-10)
Prealbumin (mg/L, normal range 200-400)	Procalcitonin (ng/ml, normal range 0-0.05)
Albumin (G/L, normal range 35-55)	Interleukin -6 (Pg/ml, normal range 0-7)
Creatine kinase (U/L, normal range Male 55-170; Female **30-135**)	CD4 T-cell (per ul, normal range 493-1191)
Creatine kinase isoenzyme (ng/ml, normal range 0.0-3.4)	CD8 T-cell (per ul, normal range 182-785)
	Total T-Cell (per ul, normal range 644-2201)
